# Money flow network among firms’ accounts in a regional bank of Japan

**DOI:** 10.1140/epjds/s13688-021-00274-x

**Published:** 2021-04-21

**Authors:** Yoshi Fujiwara, Hiroyasu Inoue, Takayuki Yamaguchi, Hideaki Aoyama, Takuma Tanaka, Kentaro Kikuchi

**Affiliations:** 1 Graduate School of Information Science, University of Hyogo, 650-0047 Kobe, Japan; 2 The Center for Data Science Education and Research, Shiga University, 522-8522 Hikone, Japan; 3 RIKEN iTHEMS, Wako, 351-0198 Saitama, Japan; 4 Research Institute of Economy, Trade and Industry, 100-0013 Tokyo, Japan; 5 Graduate School of Data Science, Shiga University, 522-8522 Hikone, Japan; 6 Graduate School of Economics, Shiga University, 522-8522 Hikone, Japan

**Keywords:** Input-output table, Hodge decomposition, Non-negative matrix factorization, Bowtie-walnut structure

## Abstract

In this study, we investigate the flow of money among bank accounts possessed by firms in a region by employing an exhaustive list of all the bank transfers in a regional bank in Japan, to clarify how the network of money flow is related to the economic activities of the firms. The network statistics and structures are examined and shown to be similar to those of a nationwide production network. Specifically, the bowtie analysis indicates what we refer to as a “walnut” structure with core and upstream/downstream components. To quantify the location of an individual account in the network, we used the Hodge decomposition method and found that the Hodge potential of the account has a significant correlation to its position in the bowtie structure as well as to its net flow of incoming and outgoing money and links, namely the net demand/supply of individual accounts. In addition, we used non-negative matrix factorization to identify important factors underlying the entire flow of money; it can be interpreted that these factors are associated with regional economic activities. One factor has a feature whereby the remittance source is localized to the largest city in the region, while the destination is scattered. The other factors correspond to the economic activities specific to different local places. This study serves as a basis for further investigation on the relationship between money flow and economic activities of firms.

## Introduction

Determining how money flows among economic entities is an important aspect of understanding the underlying economic activities. For example, the so-called flow of funds accounts record the financial transactions and the resulting credits and liabilities among households, firms, banks, and the government (see, e.g., [1]). Another example is the input-output table, which describes the purchase and sale relationships among producers and consumers within an economy and clarifies the flows of final and intermediate goods and services with respect to industrial sectors and product outputs (e.g., [2]). These data are used in macroscopic studies, such as those of industrial sectors and aggregated economic entities.

Recent years have witnessed the increasing emergence of microscopic data. For example, one can study a nationwide production network, i.e., how individual firms transfer money among one another as suppliers and customers for transactions of goods and services (see [3] and the references therein). In contrast to the macroscopic studies mentioned above, microscopic studies can uncover the heterogeneous structure of the network and its role in economic activities, how the activities are subject to shocks due to natural disasters [4] and pandemics [5], and so forth. However, microscopic data are not exhaustive; although they may cover most active firms, not all the suppliers and customers are recorded. Such records are based on a survey in which a firm nominates a selected number of important customers and suppliers. In addition, the transaction amounts are often lacking; hence, the network is directed but only binary. More importantly, microscopic and macroscopic data are compiled and updated annually or quarterly at most (see [3, 6] and the references therein).

To uncover how economic entities such as firms perform economic activities in a real economy, we should ideally study how money flows among firms by using real-time data of bank transfers with exhaustive lists of accounts and transfers. Also, investigating money flows among accounts will help to tackle real-world problems including the prediction of the economic impact of COVID-19, the defaults of firms, and the bank accounts that could be involved in illegal activities. However, these problems have been addressed without utilizing the information about the network of money flow [7]. The prediction accuracy will be improved by taking into account the network as well as other features. To the best of our knowledge, such a study has not been conducted thus far, simply because such data are not available for academic purposes. The present study precisely performs such an analysis of a Japanese bank’s dataset. The bank is a regional bank, which has a high market share with respect to the loans and deposits in a prefecture, particularly supporting financial transactions among the manufacturing firms located there (according to a disclosure issued by the bank).

The objective of this study is to investigate economic activities via bank transfers among firms’ accounts by selecting all the transfers related to the firms to uncover how money flows behind the economic activities. More specifically, we examine the network and flow structures, especially the so-called bowtie structure, to locate the position of individual accounts upstream and downstream of the entire flow. We quantify the location using the method of Hodge decomposition of the flow. Furthermore, we examine geographical information of bank transfers in order to see how geographical relations between remittance source and destination are represented by a small number of components of areas.

## Data

Our dataset comprises all the bank transfers that are sent from or received by the bank accounts in a regional bank. The regional bank is Shiga Bank, Ltd., the largest bank in a prefecture in Japan, which is mid-sized in terms of its population (more than a million) and economic activity. All the accounts are anonymous for obvious reasons, while several attributes such as geographical locations are given to the accounts owned by firms under the anonymity. Hereafter, we refer to it simply as Bank A for brevity. The period covered in our study is from March 1, 2017, to July 31, 2019, i.e., a period of 29 months or 883 days.

During this period, there were 23 million transfers among 1.7 million bank accounts involving a total of 17.4 trillion yen (roughly 160 billion USD or 140 billion Euros). Let us denote a transfer from account *i* to account *j* by $i\rightarrow j$. To focus only on the firms’ accounts in Bank A, we filtered the data such that (i) both *i* and *j* are the accounts of Bank A, (ii) both *i* and *j* are owned by firms excluding households, and (iii) self-loops $i\rightarrow i$ are deleted. Point (ii) is important for our purpose, because our concern here is how money flows and circulates among firms’ accounts, which is considered to be closely related to the firms’ economic activities. The resulting data are summarized in Table 1 (see the rightmost column).

**Table 1:** Bank accounts and transfers: summary

Number/Amount	Entire data	Within Bank A	
		All	Firms
#Accounts	1.71 M	642,411	30,613
#Transfers	23.06 M	12,847,963	2,409,619
#Links	3.13 M	1,470,107	280,864
Transfer (Yen)	17.43 T	5.26 T	2.15 T

For a transfer *i*→*j*, the column “Entire data” includes the cases in which either *i* or *j* is not an account of Bank A. The column “Within Bank A” corresponds to the case in which both *i* and *j* are accounts of Bank A. “firms” implies that both the source and the target of a link are firm accounts. M and T denote million and trillion, respectively.

**Figure 1:**
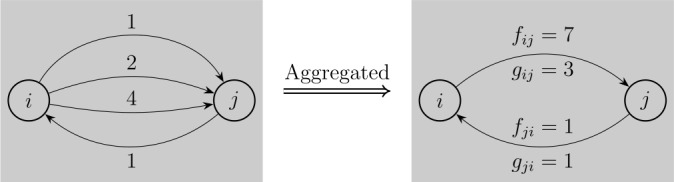
Construction of bank-transfer network by aggregation. How bank transfers are aggregated into links. *i* made three transfers (1, 2, and 4) in an arbitrary unit of money to *j*, while *j* made one transfer (1) to *i* during a certain period. Flow $f_{ij}$ is defined by the total flow of transfers along $i\rightarrow j$. Frequency $g_{ij}$ is the frequency of these transfers

Note that multiple transfers $i\rightarrow j$ can exist for a given pair of *i* and *j*, because of frequent transfers. One can quantify the strength of the directional relationship between a pair of accounts either by the flow of transfers or by their frequency. To do so, we aggregate multiple transfers, if present, into a single link $i\rightarrow j$ with two types of weights, namely flow $f_{ij}$ and frequency $g_{ij}$ (see the illustration in Fig. 1). Hereafter, we use the term *link* for aggregated transfers.

The number of accounts or nodes in the network is $N=30{,}613$, while the number of links is $M=280{,}864$ after the aggregation (see Table 1).

The summary statistics of the links’ flows $f_{ij}$ and frequencies $g_{ij}$ for all the pairs of accounts *i* and *j* are presented in Table 2. One can observe that the distributions for flow and frequency have large skewness, implying that a considerable fraction of the money flow is due to a large amount transferred by a small number of flows.

**Table 2:** Summary statistics for links’ flows and frequencies

Stats.	Flow (Yen)	Frequency
Min.	1	1
Max.	$ 3.00\times10^{10} $	2,616
Median	$ 0.20\times10^{6} $	3
Avg.	$ 7.65\times10^{6} $	8.58
Std.	$ 1.53\times10^{8} $	19.92
Skewness	92.5	37.8
Kurtosis	$ 1.25\times10^{4} $	$ 3.49\times10^{3} $

Summary statistics of the links’ flows and frequencies for all the pairs of accounts, where links are aggregated transfers as defined in the main text and Fig. 1.

## Results and discussion

### Network of firms’ accounts and links of transfers

**Figure 2:**
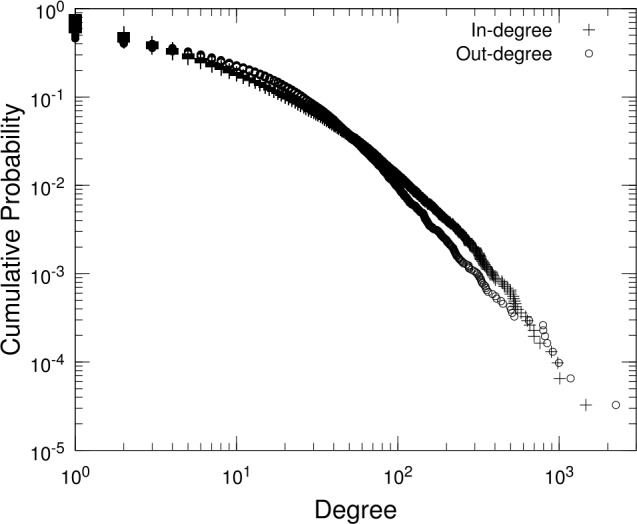
Degree distributions for the bank transfer network. Complementary cumulative distributions for in-degree and out-degree, which refer to the number of incoming and outgoing links, respectively, of each account

First, let us summarize the network structure comprising firms’ accounts as nodes and aggregated transfers as links. We remark that transfers are aggregated into links as shown in Fig. 1. The degree is the number of transfers received by or sent from an account. The number of incoming and outgoing links of an account is called the in-degree and out-degree, respectively. Figure 2 shows the distributions of the in-degree and out-degree as complementary cumulative distributions. By noting that the total number of accounts is $N=30{,}613$, we can see that a small fraction of accounts has a considerable degree, i.e., a thousand or more links, while most accounts have a limited number of links. In fact, the tail in Fig. 2 can be approximated by a Pareto distribution $P(k)\propto k^{-\mu }$ for degree *k* with an exponent *μ*, which can be estimated as $\mu =1.63(\pm 0.05)$ for in-degree and $\mu =1.99(\pm 0.06)$ for out-degree (standard errors in parentheses), both obtained by Hill’s estimator for the top 1000 (corresponding to roughly $k>50$). Such hubs are presumably entities associated with the local government or the public sector in the region. We summarize the basic properties of the network in Appendix A.

**Figure 3:**
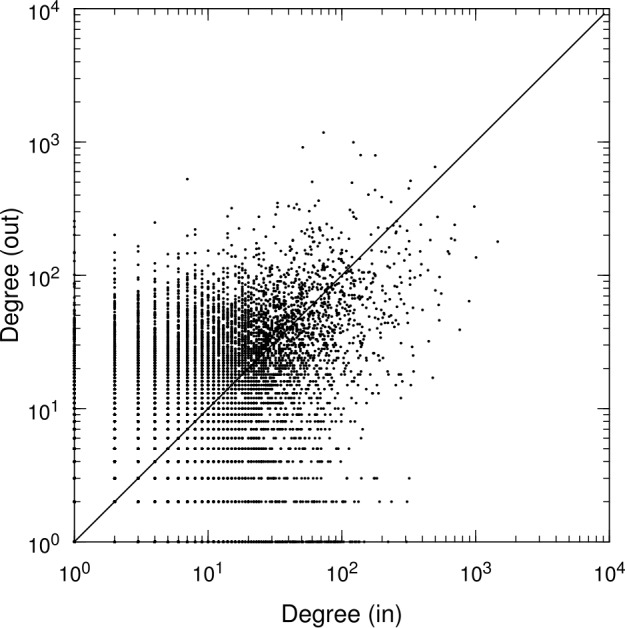
Scatter plot for in-degree and out-degree of each account. Each account as a node, represented as a point, has incoming links and outgoing links, the numbers of which are represented by the horizontal and vertical axes, respectively. The diagonal line represents the locations where the in-degree and out-degree are equal

Because each node has an in-degree and out-degree, we can examine how they are correlated. Figure 3 shows the scatter plot for the in-degree and out-degree of each account. We can observe a tendency for a positive correlation between the degrees (Pearson’s $r=0.303$ ($p<10^{-6}$); Kendall’s $\tau =0.164$ ($p<10^{-6}$)). We also observe the accounts that have many more incoming links than the outgoing ones (and vice versa), which can be respectively considered as “sinks” and “sources” with respect to money flow. If household accounts were included, one would have a larger number of sinks corresponding to the situation that income and saving are likely larger than expenditure and dissaving, but such sinks are not present here.

**Figure 4:**
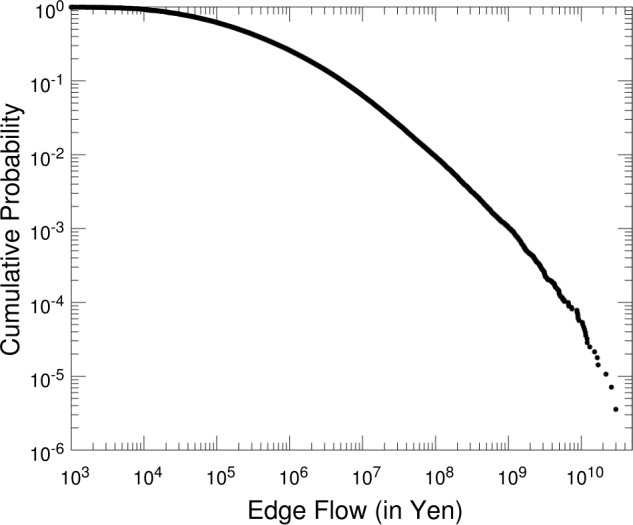
Distribution for the flows of links. Complementary cumulative distributions for the amount of money defined by $f_{ij}$ between each pair of accounts *i* and *j* (see Fig. 1)

We can observe each link’s weights, flow $f_{ij}$, and frequency $g_{ij}$ (see Fig. 1). Figure 4 shows the complementary cumulative distribution for the flow along each link. The distribution is highly skewed; there exist a small number of links that have a large amount of flow exceeding a billion yen — likely important channels with large flows of money. Quantitatively, 0.1% of the links have flows larger than a billion yen.

**Figure 5:**
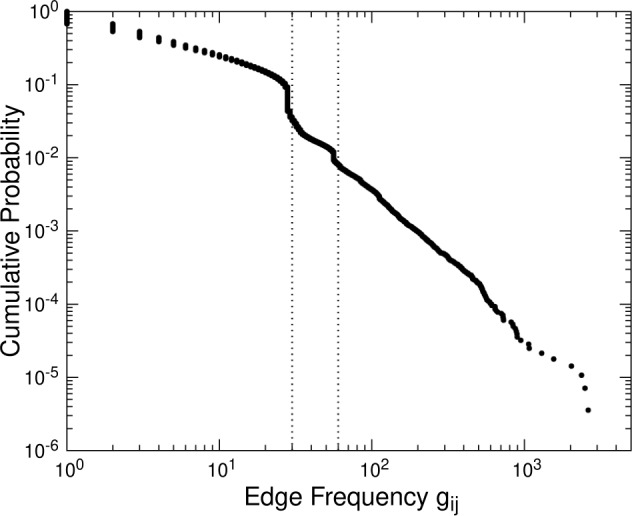
Distribution for the frequencies of transfers. Complementary cumulative distributions for the frequency defined by $g_{ij}$ between each pair of accounts *i* and *j* (see Fig. 1). We can observe that there are frequency steps around 30 and 60 (vertical dotted lines), which are presumed as periodic transfers performed once or twice in each month (recall that the entire period includes 29 months)

Figure 5 shows the complementary cumulative distribution for the frequency along each link. The steps at 30 and 60 on the horizontal axis are considered to correspond to transfers performed once or twice in each month (recall that the entire period includes 29 months). We can see that 0.1% of the links have frequencies of 500 or more corresponding to daily transfers on weekdays.

### Community analysis

Communities or clusters in a network are tightly knit groups with high intra-group density and low inter-group connectivity [8]. Community analysis is useful for understanding how a network has such heterogeneous structures. We adopt the widely used Infomap method [9, 10] to detect communities in our data.

The results are presented in Table 3. “Level” indicates the level of communities in a hierarchical tree of communities that are detected recursively (see [10]). The number of communities indicates how many communities are detected at the corresponding level. The label “irr. comm.” denotes irreducible communities that cannot be decomposed further to the next level of smaller communities in the hierarchical decomposition. For example, 143 of 164 communities at the first level are irreducible ones, whereas the rest of them are decomposed into 2327 smaller communities at the next level, and so forth.

**Table 3:** Numbers of communities, irreducible communities, and accounts at each level of community analysis using Infomap

Level	#comm.	#irr. comm.	#accounts	Ration (%)
1	164	143	355	0.012
2	2327	2264	28,948	94.5
3	215	215	1310	0.043
Total	–	2621	30,613	100.0

Each level corresponds to the hierarchical level in the Infomap community analysis [10]. A community at a level can be decomposed at the next lower level (from top to bottom). If a community cannot be decomposed further, it is called an irreducible community. The numbers of irreducible communities are listed in the third column. The fourth column lists the numbers of accounts belonging to these irreducible communities at each level.

We find that most of the communities are at the second level because of the number of accounts, and that most of the accounts (94.5%) belong to the second-level communities. In our previous study [11] on the application of hierarchical community analysis using Infomap to a large-scale production network, we showed that a relatively shallow hierarchy can be observed at the fifth level as the lowest level; in particular, most firms are included at the second level, exactly as we find here. This is reasonable, because our data on bank transfers among firms’ accounts should reflect a regional fraction of the entire production network on a nationwide scale. The finding here is interesting, because this implies a self-similar structure of the production network meaning that a partial system has a similar network property to the global system.

**Figure 6:**
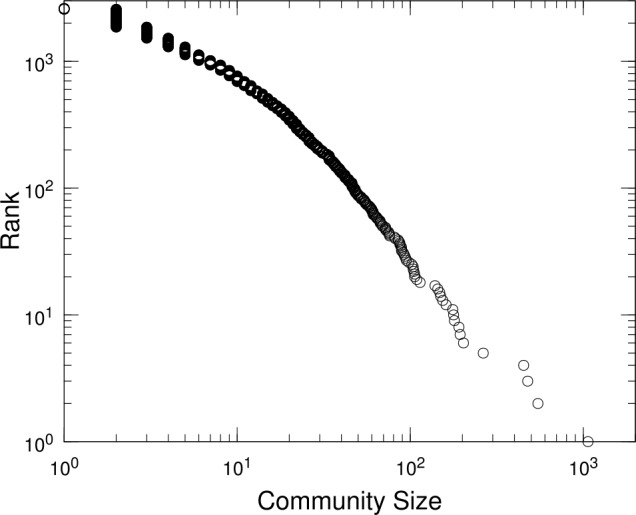
Distributions of the sizes of irreducible communities. Rank-size plot for the sizes of irreducible communities detected using the Infomap method at all the levels, where the ranks are in descending order of the size with the lowest rank equal to the total number of irreducible communities (see Table 3). The size of a community is simply the number of nodes included in the community

Figure 6 shows the distribution of the sizes of irreducible communities at the lowest level that includes all the accounts. The size of a community is simply the number of nodes included in the community. The result indicates that the size of the communities is highly skewed over a few orders of magnitude. We note that there exist more than 10 communities with sizes exceeding 100, which correspond to important clusters of economic activities that depend on geographical sub-regions and industrial sectors. We shall discuss this issue in our analysis of non-negative matrix factorization later.

### Bowtie-walnut structure

With respect to the flow of money, the accounts can be located in a classification of the so-called *bowtie* structure, which was first adopted in the study of the Internet [12]. In the context of economics and finance, the method has been applied to business relationship networks [13] and credit default swap network [14], for example. Nodes in a directed network can be classified into a giant strongly connected component (GSCC), its upstream side as the IN component, its downstream side as the OUT component, and the rest of the nodes that do not belong to any of GSCC, IN, and OUT. In general, they can be defined as follows.

GWCC: Giant weakly connected component: the largest connected component when viewed as an undirected graph. At least one undirected path exists for an arbitrary pair of nodes in the component.
GSCC: Giant strongly connected component: the largest connected component when viewed as a directed graph. At least one directed path exists for an arbitrary pair of nodes in the component.
IN: Nodes from which the GSCC is reached via directed paths.
OUT: Nodes that are reachable from the GSCC via directed paths.
TE: “Tendrils”: the rest of GWCC.

Therefore, we have the following decomposition of GWCC:

1 $$ \text{GWCC}=\text{GSCC}+\text{IN}+\text{OUT}+\text{TE} . $$

**Figure 7:**
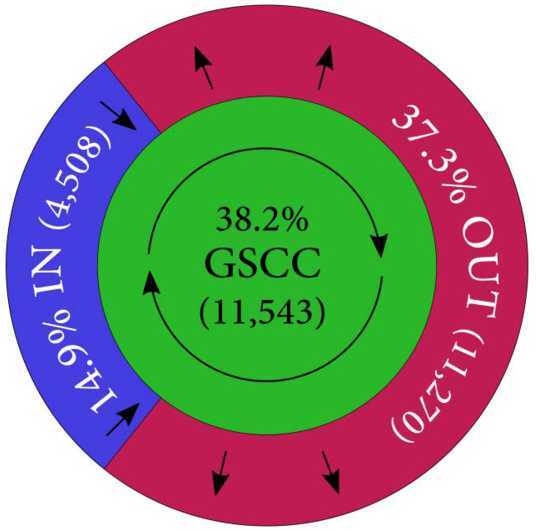
Walnut structure: a schematic view. The so-called bowtie structure reveals that GSCC includes nearly 40% of all the nodes or accounts, while the IN and OUT components include 15% and 37%, respectively (see Table 4 for the details). The prominent features are as follows. (i) The shortest distances to IN and OUT from GSCC are quite small, typically 1 or 2, and 4 at most (Table 5); hence, the ties are not elongated like a “bowtie” but rather like a “walnut” skin. (ii) The nodes in the components of IN and OUT are connected to the nodes scattered widely in GSCC. See also the study of a supplier-customer network [11] with similar features

For our data of the entire network with $N=30{,}613$ nodes and $M=280{,}864$ links, the GWCC component comprises 30,225 (99.0%) nodes and 280,598 (99.9%) links. The breakdown of GWCC to GSCC, IN, OUT and TE is given in Table 4. As is seen here, nearly 40% of the accounts are inside GSCC. Further, 15% of the accounts are in the upstream portion or IN, whereas 37% are in the downstream portion or OUT (see Fig. 7). These figures are similar to those observed in the production network in Japan in a previous study [11].

**Table 4:** Bowtie or “walnut” structure: size of each component

Component	#accounts	Ratio (%)
GSCC	11,543	38.2%
IN	4508	14.9%
OUT	11,270	37.3%
TE	2904	9.6%
Total	30,225	100%

“Ratio” refers to the ratio of the number of firms to the total number of accounts in GWCC.

The similarity between the current money-flow network and the production network requires careful elaboration. First, the flow in the current network is in the direction of money transfer, while in the production network the flow is in the direction of goods and services, i.e., from suppliers to customers. Therefore, the IN component in the production network should be compared to the OUT component and vice versa. Upon making this comparison, we notice that the OUT component in the current network occupies a much larger portion of the network (37.3%) than the one in the production network (20.6%; see [11]). This is understandable based on two facts: (i) nodes are bank accounts in the current network, while in the production network, nodes are firms’ headquarters; (ii) the prefecture where the current regional bank resides is void of major cities of Japan, such as Tokyo, Osaka or Nagoya. This implies that many firm headquarters are outside of this prefecture and the prefecture is dominated by agriculture and production facilities. Given that the number of bank accounts of factories and associated offices is expected to be much higher than that of accounts being closer to consumer market, the OUT component in the current network occupies a larger portion than it does in the production network.

The global structure of the network, its connectivity, is an another important property. The term “bowtie” refers to the connectivity structure observed in many social and technological networks, such as the Internet, where the maximum distances from GSCC to IN or OUT are often very long (see the original paper [12]) and in fact look similar to a bowtie in their visualization. However, in the case of production networks, it was found that the connectivity between IN and GSCC and the one between GSCC and OUT is very high: Over 90% of nodes in IN can reach a node in GSCC with only one link and similarly with from OUT to GSCC. Here, network visualization showed a tightly bound shape, where IN and OUT forms two thin half shells with GSCC at the core, reflecting the high connectivity. The authors of [11] found that this structure, lacking two wings elongating from the center, does not resemble a “bowtie” at all and coined the more fitting term “walnut” to describe it. The shortest-path lengths between GSCC and IN or OUT in the current money-flow network is given in Table 5, where we can observe that the accounts in the IN and OUT components are only a few steps away from GSCC: the money-flow network is “walnut” in structure, similar to the production network. This is most understandable when we look at their relationship. Given that firm headquarters form a tight “walnut” network, their factories and related offices cannot form an elongated link structure.

**Table 5:** “Walnut” structure: shortest distance from GSCC to IN/OUT

IN to GSCC			OUT from GSCC		
Distance	#accounts	Ratio(%)	Distance	#accounts	Ratio(%)
1	4346	96.41%	1	11,051	98.06%
2	144	3.19%	2	208	1.85%
3	8	0.18%	3	11	0.10%
4	10	0.22%	4	0	0.00%
Total	4508	100%	Total	11,270	100%

The left half lists the number of accounts in the IN component connected to the GSCC accounts with the shortest distances within 4 at most. The right half represents the OUT component similarly.

Finally, it should be noted that the decomposition of IN, OUT, and GSCC components is based on the identification of the largest strongly connected component (GSCC) and reachability to it from other part of the network. Individually, there is no significant difference in each network structure. Each component is merely a subgraph of the original network; a part of bank transfers reflecting the supplier-customer relationship of firms. The difference is the relative position of IN and OUT with respect to GSCC, which can be quantified and interpreted as relative position in the upstream and downstream of money flow (as discussed in the following section).

### Hodge decomposition: upstream/downstream flow

Our analysis of the bowtie structure implies that the nodes in IN and OUT are located in the upstream and downstream sides in the flow of money. The Hodge decomposition of the flow in a network is a mathematical method of ranking nodes according to their locations upstream or downstream of the flow [15]. This method, also known as the Helmholtz–Hodge–Kodaira decomposition, has been used to find such a structure in complex networks (see, e.g., neural networks [16] and economic networks [17–19]).

First, we recapitulate the method in a manner suitable for our purpose here. Let $A_{ij}$ denote adjacency matrix of our directed network of bank transfers, i.e.,

2 $$\begin{aligned} A_{ij} &= \textstyle\begin{cases} 1 & \text{if there is a link of transfer from account }i\mbox{ to }j, \\ 0 & \text{otherwise}. \end{cases}\displaystyle \end{aligned}$$

Recall that the numbers of accounts and links are *N* and *M*, respectively. We excluded all the self-loops, implying that $A_{ii}=0$. Each link has a flow, denoted by $\tilde{F}_{ij}$, either of the total amount of transfers, $f_{ij}$, or the frequency of transfers, $g_{ij}$ (see Fig. 1), i.e.,

3 $$\begin{aligned} \tilde{F}_{ij} &= \textstyle\begin{cases} f_{ij} \text{ or } g_{ij} & \text{if }A_{ij}=1, \\ 0 & \text{otherwise} . \end{cases}\displaystyle \end{aligned}$$

Note that there may be a pair of accounts such that $A_{ij}=A_{ji}=1$ and $\tilde{F}_{ij}, \tilde{F}_{ji}>0$. Next, we shall take the frequency of transfers, $g_{ij}$, by assuming that it represents the strength of the link.

Let us define a “net flow” $F_{ij}$ by

4 $$ F_{ij}=\tilde{F}_{ij}- \tilde{F}_{ji} $$

and a “net weight” $w_{ij}$ by

5 $$ w_{ij}=A_{ij}+A_{ji}. $$

Note that $w_{ij}$ is symmetric, i.e., $w_{ij}= w_{ji}$, and non-negative, i.e., $w_{ij}\geq 0$ for any pair of *i* and *j*. We remark that Eq. (5) is simply a convention to consider the effect of mutual links between *i* and *j*. One could multiply Eq. (5) by 0.5 or an arbitrary positive number, which does not change the result significantly for a large network.

Now, the Hodge decomposition is given by

6 $$ F_{ij}=F^{(\text{c})}_{ij}+F^{(\text{g})}_{ij}, $$

where the *circular flow* $F^{(\text{c})}_{ij}$ satisfies

7 $$ \sum_{j} F^{(\text{c})}_{ij}=0, $$

which implies that the circular flow is divergence-free. The *gradient flow* $F^{(\text{g})}_{ij}$ can be expressed as

8 $$ F^{(\text{g})}_{ij}=w_{ij}(\phi _{i} - \phi _{j}), $$

i.e., the difference of “potentials”. In this manner, the weight $w_{ij}$ serves to make the gradient flow possible only where a link exists. We refer to the quantity $\phi _{i}$ as the *Hodge potential*. If $\phi _{i}$ is relatively large, the account *i* is located in the upstream side of the entire network, while a small $\phi _{i}$ implies that *i* is located in the downstream side of the entire network.

Equations (6)–(8) can be solved as follows. First, we combine them into the following equation for the Hodge potentials $(\phi _{1},\ldots ,\phi _{N}) (\equiv \boldsymbol{\phi })$:

9 $$ \sum_{j} L_{ij} \phi _{j} = \sum_{j} F_{ij} , $$

for $i=1,\ldots ,N$. Here, $L_{ij}$ is the so-called graph Laplacian and defined by

10 $$ L_{ij}=\delta _{ij} \sum _{k} w_{ik} - w_{ij} , $$

where $\delta _{ij}$ is the Kronecker delta.

It is straightforward to show that the matrix $L=(L_{ij})$ has only one zero mode (eigenvector with zero eigenvalue), i.e., $\boldsymbol{\phi }=(1,1,\ldots ,1)/\sqrt{N}$. The presence of this zero mode simply corresponds to the arbitrariness in the origin of *ϕ*. We can show that all the other eigenvalues are positive (see, e.g., [20]). Therefore, Eq. (9) can be solved for the potentials by fixing the potentials’ origin. We assume that the average value of *ϕ* is zero, i.e., $\sum_{i} \phi _{i}=0$.

We note that the Hodge decomposition described above plays an essential role in deciphering structure of the entire network, as well as the position and the role of each nodes in it. In studying the nodes, one may think of simply evaluating the cumulative out-flows and use it in place of the Hodge potential. This, however, misses the whole point of studying the network: Let us think of two nodes in the IN component, who have the same total out-flow. If we use the total out-flow as a measure of their locations, they are at an equal level, regardless of to whom they are connected: even if one is connected to a GSCC node close to the IN side and the other is connected to a GSCC node close to the OUT side. This also applies to those GSCC nodes in a reverse way: in evaluating the location of those GSCC nodes it is important to whom in the IN/OUT component they are connected. The Hodge decomposition solves this problem at once, as it is based on the network structure. Those IN nodes will be given appropriate Hodge potential in relation with their connection to those GSCC nodes, who again are given appropriate Hodge potential with view of all the other edges of the entire network. (See Appendix B for some intuitive explanation and simple examples.)

**Figure 8:**
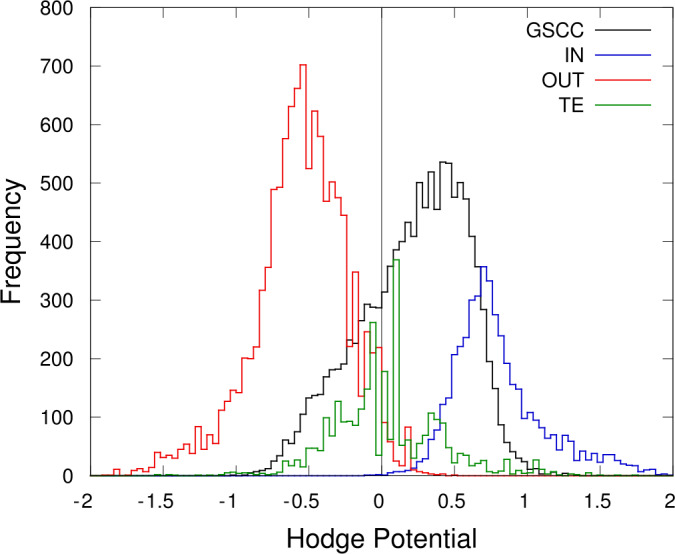
Distribution of the Hodge potentials of individual accounts. Distributions as histograms of $\phi_{i}$ in each component of the bowtie or walnut structure Fig. 7. The horizontal axis represents the value of $\phi_{i}$ of an individual node or account, while the vertical axis represents the frequency in the histogram. The black line corresponds to GSCC or the core. The blue and red lines, respectively, correspond to the IN and OUT components or upstream and downstream with respect to the core. The green line corresponds to TE (tendrils) or the rest of the nodes

The Hodge potentials obtained for the entire network of GWCC are shown in Fig. 8 as the distribution for the potentials of all the accounts in GWCC. By noting that the average is zero by definition, we can see that it is a bimodal distribution with two peaks at positive and negative values, while there are a number of potential values close to zero (peaks around zero). The nodes in TE (tendrils) can be considered to have locations that are not particularly relevant to upstream or downstream; we can expect that these nodes mostly have potentials close to zero, as shown by the green line, i.e., the result after deleting all the nodes contained in TE’s. We can see that these TE do not contribute to large absolute values of the Hodge potentials.

**Figure 9:**
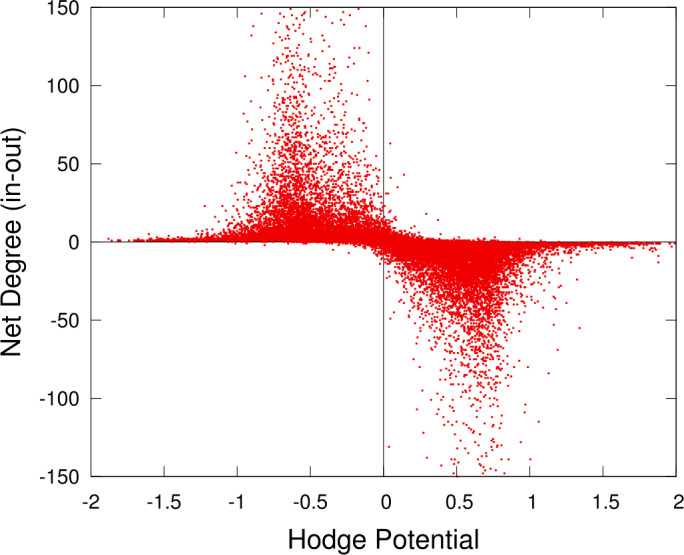
Hodge potential and net degree for each node. Each point represents a node or an account. The net degree is defined by the difference between the in-degree and the out-degree of the node. If the net degree is positive, the node has more incoming links than outgoing ones and vice versa

**Figure 10:**
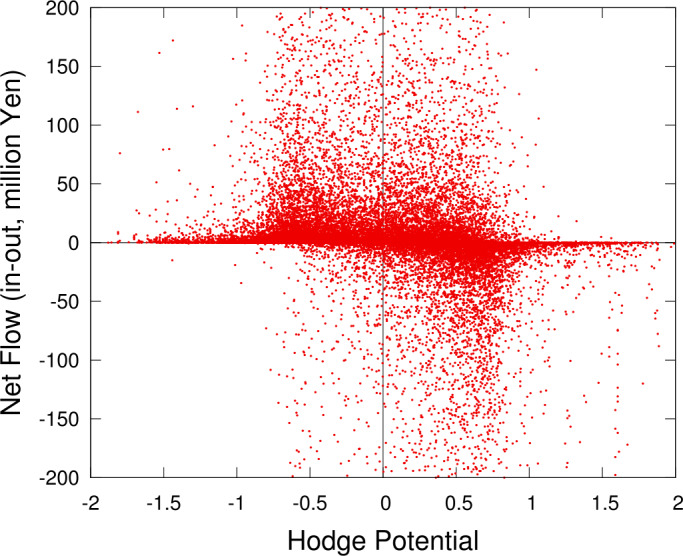
Hodge potential and net flow for each node. This figure is similar to Fig. 9 except for the vertical axis, which represents the net flow. The net flow is defined by the difference between the incoming amount of money and the outgoing one

It can be expected that there is a correlation between the value of the Hodge potential and the *net* amount of demand or supply of money for each node. We can measure the net amount of demand/supply by examining the in-degree and out-degree of the node, or alternatively, the in-flow and out-flow of money. Figure 9 and Fig. 10 show the results. We find that if the potential is positive, the node is located in the upstream side, and its net degree and flow are negative. If the potential is negative, the node is located in the downstream side, and its net degree and flow are positive.

This finding can be interpreted as follows. Consider a supplier in the production network, which supplies its products to a number of customers. The supplier has a bank account (or possibly multiple accounts) that receives money from the customers’ accounts as the supplier’s sales. If the supplier is in the upstream side of the supplier-customer relationship, it is likely that the account is located in the downstream side of the money flows in this study. As the supplier not only makes sales but also incurs costs, typically labor costs, there must be an outgoing flow from its account to be linked with households and other non-commercial entities, which are not included in the present study. Consequently, the supplier’s account has a positive net degree and flow, while its Hodge potential is likely negative. A similar argument would hold for customers in an opposite way. In other words, our finding is a direct observation of how the flow of money reflects the economic activities among the firms’ accounts.

In response, a keen reader may wonder how the results might change if the consumption of households is included in our study. In fact, in case of the economic activity of households in this particular region as well as other regions of Japan during the period of our study, cash was the largest channel of payment. Hence, transfers from households to firms are quite negligible in frequency and amount, even if the consumption of households is included.

### Non-negative matrix factorization (NMF): decomposition of geographical structures of bank transfers

In this section, we focus on the geographical information of bank transfers. Each bank account has a registered address, when the account was created. We obtain the latitudes and longitudes of the bank accounts by using geocoding. Consequently, a bank transfer between two bank accounts has two coordinates of its remittance source and destination. Can geographical relations between source and destination be represented by only a small number of components of areas? We construct a non-negative matrix defined from the frequencies between the geographical areas, and we adopt NMF to find such components of geographical structures of the bank transfers.

NMF constructs an approximate factorization of a non-negative matrix [21]. Applications of NMF to real dataset give a small number of components whose linear sums can approximate elements of the dataset. For example, NMF is useful for processing facial images because it produces parts-based representations of such images [22]. To obtain the basic components whose linear sums can approximate bank transfers, we apply NMF to a non-negative matrix *V* constructed as a geographical aggregation from the frequencies of bank transfers $g_{ij}$ in the following way.

Let the geographical location of account *i* be $\operatorname{loc}(i)$, that is, the pair of the longitude and latitude of the registered address of *i*. We set a lattice grid in the entire region including the Shiga prefecture using *L* by *L* sufficiently small squares, where $L=100$. Let $R_{\ell }$ be such squares ($\ell =1,2,\ldots ,L^{2}$). Aggregate the frequencies of bank transfers from a source grid $R_{s}$ to a destination grid $R_{d}$ by

11 $$ \tilde{g}_{sd}=\sum_{\{(i,j)\mid \operatorname{loc}(i)\in R_{s} \text{ and }\operatorname{loc}(j)\in R_{d}\}} g_{ij}, $$

where the summation is taken over all pairs of accounts $(i,j)$ such that the source *i* is located in $R_{s}$ and the destination *j* is located in $R_{d}$. Then let us convert the aggregated frequency to its logarithm to reduce the influence of outstanding values by

12 $$ V_{sd}=\log \bigl(\max \{1, \tilde{g}_{sd}\}\bigr) . $$

Note that every entry $V_{sd}$ is non-negative. $V=(V_{sd})$ is a sparse matrix of size $L^{2}\times L^{2}$; that is, only a small fraction of the entries are non-zero because bank transfers do not occur between many pairs of source and destination, for which we have $V_{sd}=0$.

NMF provides the approximate factorization:

13 $$\begin{aligned} V \approx WH, \end{aligned}$$

where *W* and *H* are non-negative matrices of size $L^{2}\times K$ and $K\times L^{2}$ respectively and *K* is an integer. Because of the sparsity of *V*, we expect that $K\ll L^{2}$. We assume that the approximation is based on the minimization of the following loss function given by the Frobenius norm:

14 $$ f(W,H) = \frac{1}{2}\sum _{s,d}\bigl(V_{sd}-(WH)_{sd} \bigr)^{2},\quad \text{where }W\geq 0\mbox{ and }H\geq 0, $$

where $W\geq 0$ and $H\geq 0$ implies non-negativity. Technically, we solve Eq. (14) numerically with the initialization of *W*, *H* using non-negative double singular value decomposition (see the review [23] and references therein). The minimization yields local minima in general. However, our numerical solutions under different random seeds provided essentially the same decomposition.

The decomposition by NMF can be interpreted as follows. Equation (13) is explicitly written as

15 $$ V_{sd}\approx \sum_{k=1}^{K} W_{sk} H_{kd} . $$

For an arbitrary source *s*, Eq. (15) can read as

16 $$ V_{s\bullet }\approx \sum_{k=1}^{K} W_{sk} h_{k} , $$

where $h_{k}$ is the vector given by the *k*th row of *H*. This equation means that the transfers from the source *s* can be expanded by such “basis vectors” $h_{k}$ ($k=1,2,\ldots ,K$). The basis vector’s components $(h_{k})_{d}=H_{kd}$ represent a spatial pattern of how *destinations* *d* are distributed geographically for the *k*th NMF component. Similar to an arbitrary destination *d*, one has

17 $$ V_{\bullet d}\approx \sum_{k=1}^{K} H_{kd} w_{k} , $$

where $w_{k}$ is the vector given by the *k*th column of *W*. This implies that the transfers to the destination *d* can be expanded by the basis vectors $w_{k}$ ($k=1,2,\ldots ,K$). The basis vector’s components $(w_{k})_{s}=W_{sk}$ represent a spatial pattern of how *sources* *s* are distributed geographically for the *k*th NMF component. In fact, we can regard Eq. (13) as the approximation of *V* by the sum of products of these basis vectors:

18 $$\begin{aligned} V & \approx \sum_{k=1}^{K} w_{k} h_{k}. \end{aligned}$$

This expression can be understood in the way that bank transfers can be decomposed into *K* “NMF components” comprising pairs of basis vectors, $w_{k}$ and $h_{k}$ ($k=1,2,\ldots ,K$). We let $K = 10$ from the prior knowledge that the number of local communities in the prefecture is around 10. We later discuss how results depend on different choices of *K*.

**Figure 11:**
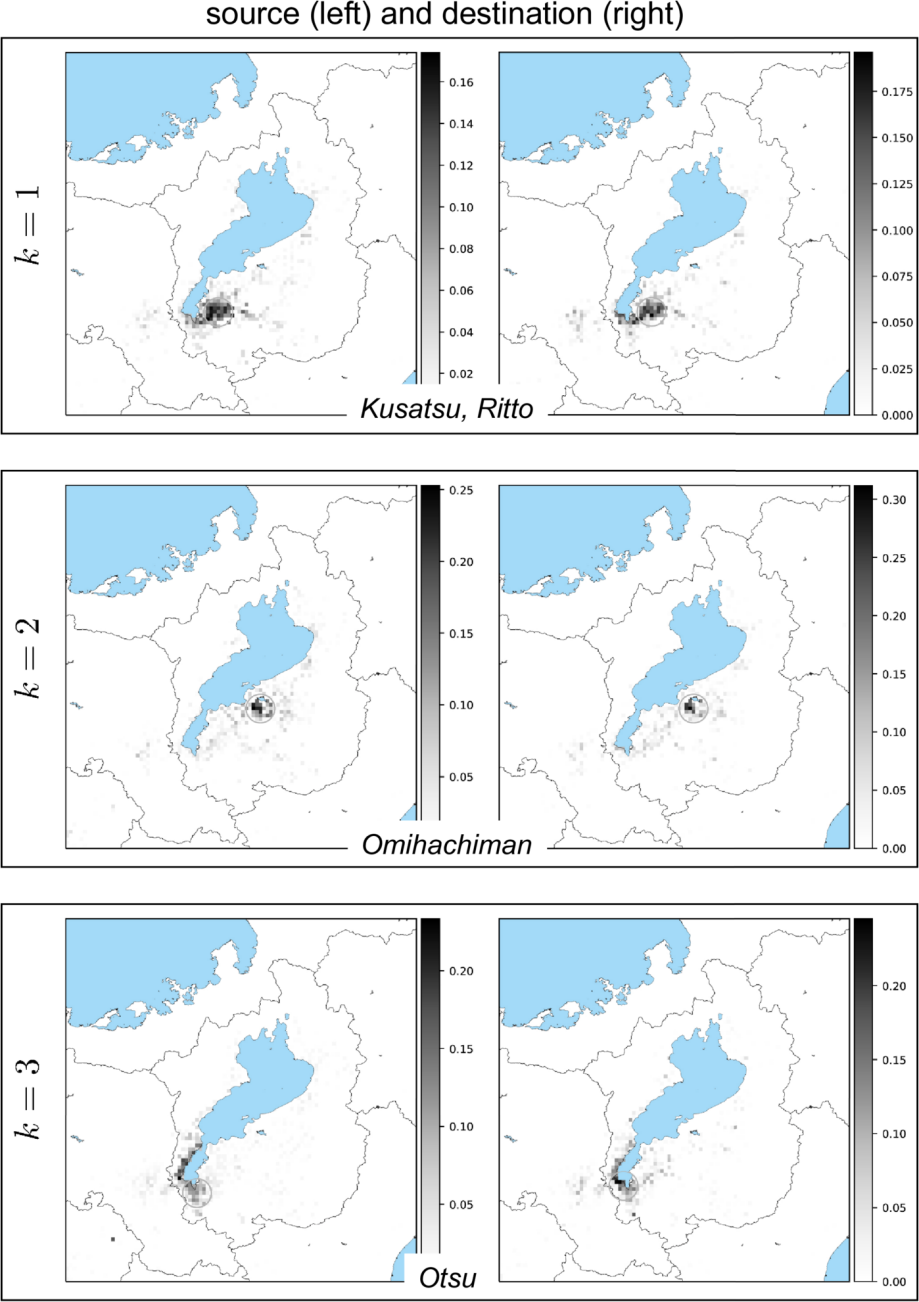
Each box shows a pair of NMF basis vectors $w_{k}$ (left) and $h_{k}$ (right). From top to bottom: $k=1,2,3$. In each box, the left figure shows the vector components of $w_{k}$ mapped onto a geographical map (lake and sea shown in blue), which represent how sources of the *k*th NMF component are distributed spatially, while the right figure shows the components of $h_{k}$, which represent how destinations of the *k*th NMF component are distributed spatially. The grayscale colorbar in each figure indicates magnitudes of the corresponding basis vector’s components after normalizing the vector by its Euclidean norm. Also shown in each figure is a small circle which corresponds to the maximum vector component. City name in each box was identified with the location of the circle

**Figure 12:**
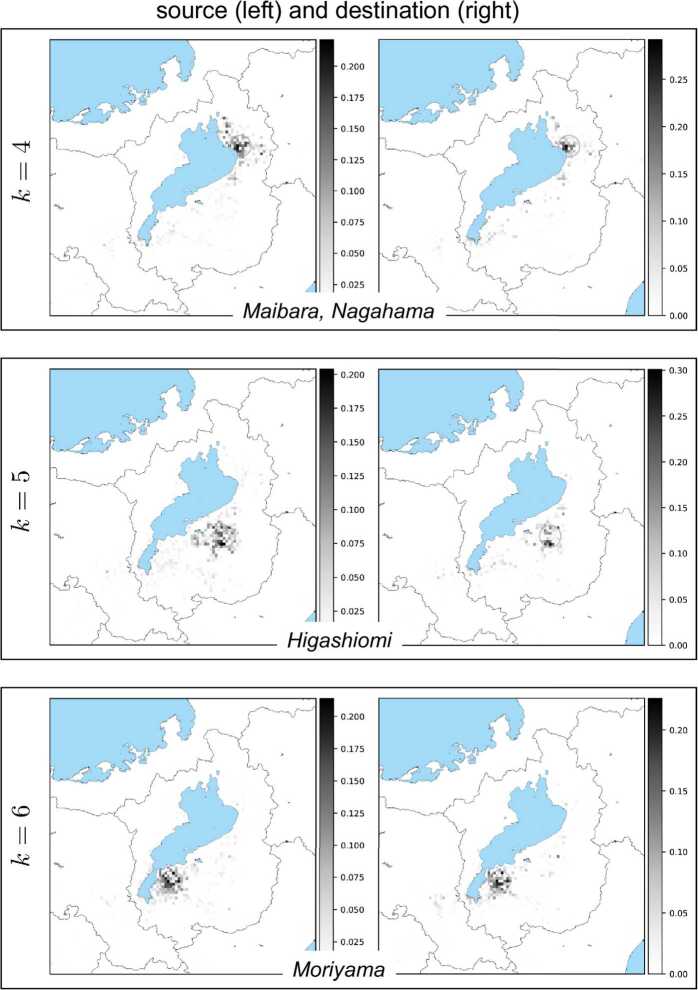
Each box shows a pair of NMF basis vectors $w_{k}$ (left) and $h_{k}$ (right). From top to bottom: $k=4,5,6$. See the caption of Fig. 11 for explanation

**Figure 13:**
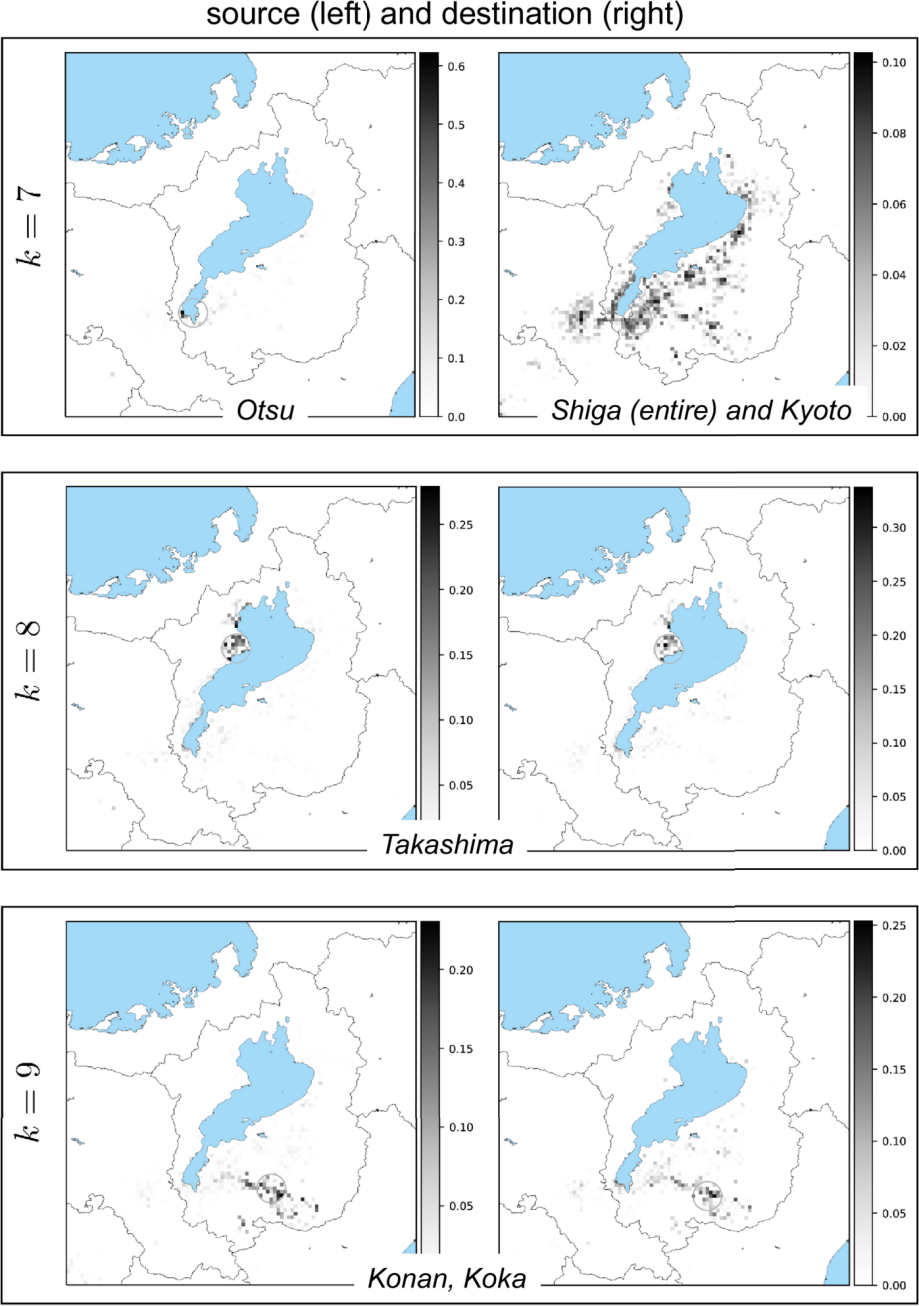
Each box shows a pair of NMF basis vectors $w_{k}$ (left) and $h_{k}$ (right). From top to bottom: $k=7,8,9$. See the caption of Fig. 11 for explanation. Note that for the box $k=7$, the basis $h_{k=7}$ (right figure) has its vector components of destination widely distributed over the Shiga prefecture and its neighbor in Kyoto, which is located to the left (west) of Shiga. So, in this exceptional case, the figure does not include a circle corresponding to any peak of vector components

**Figure 14:**
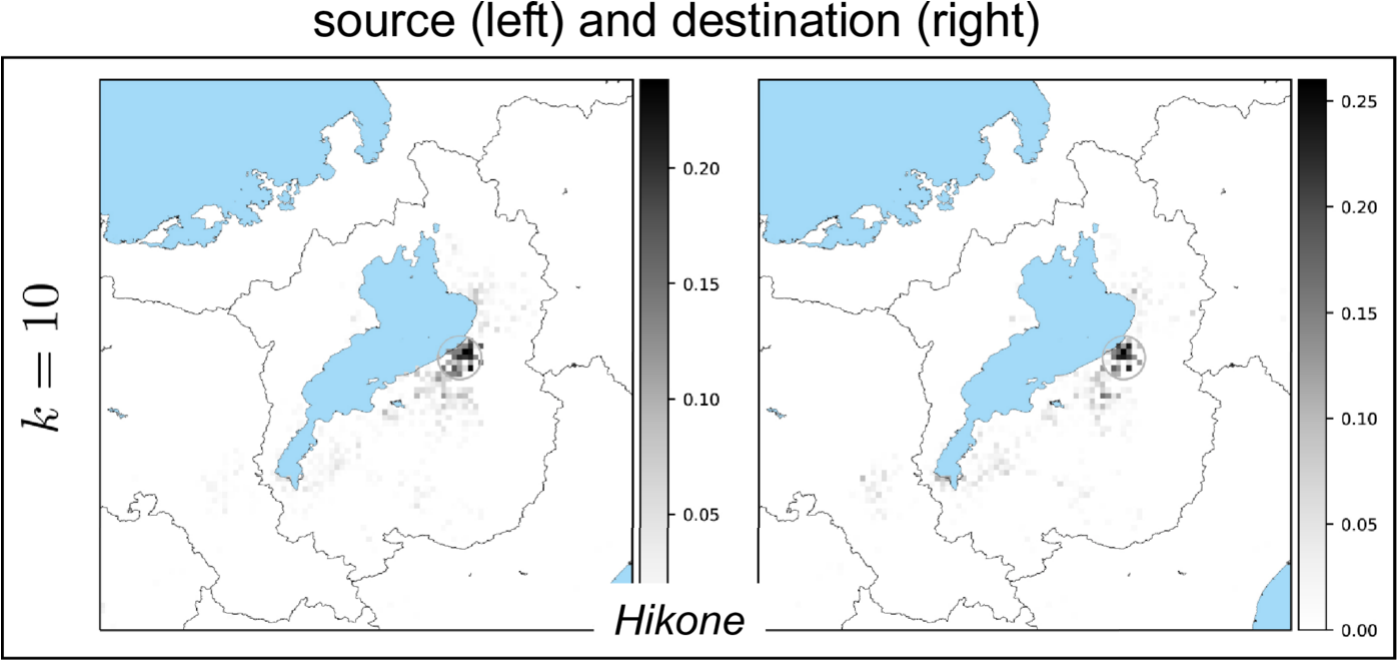
The box shows the pair of NMF basis vectors $w_{k}$ (left) and $h_{k}$ (right) for $k=10$. See the caption of Fig. 11 for explanation

Results of all the basis vectors for each NMF component $k=1,2,\ldots ,K$ are depicted in Fig. 11, Fig. 12, Fig. 13, and Fig. 14. In these figures, each box corresponds to an NMF component comprising a pair of basis vectors $w_{k}$ and $h_{k}$. Each basis vector’s components are depicted on a geographical map covering the Shiga prefecture and its surroundings including Kyoto. Larger values of vector components are indicated by darker pixels shown on the map. We can observe that the source (left figure in each box) and the destination (right) are concentrated in small geographical regions (shown by circles in each figure) due to the result that the vector components have peaks in most cases. We found that these peaks correspond to cities and highly populated urban areas. This finding holds for all *k* except one case ($k=7$). We quantified the concentration and identified the peaks. For details of the quantification and identification, see Appendix C.

The exceptional case is the basis vector $h_{k}$ for $k=7$ in Fig. 13. In this case, while the source is concentrated in the largest city of the Shiga prefecture, the destination spreads over the entire prefecture and also its neighboring city of Kyoto. This implies that one of the NMF components corresponds to bank transfers from firms in the largest city to other firms in different local areas as well as in Kyoto.

**Figure 15:**
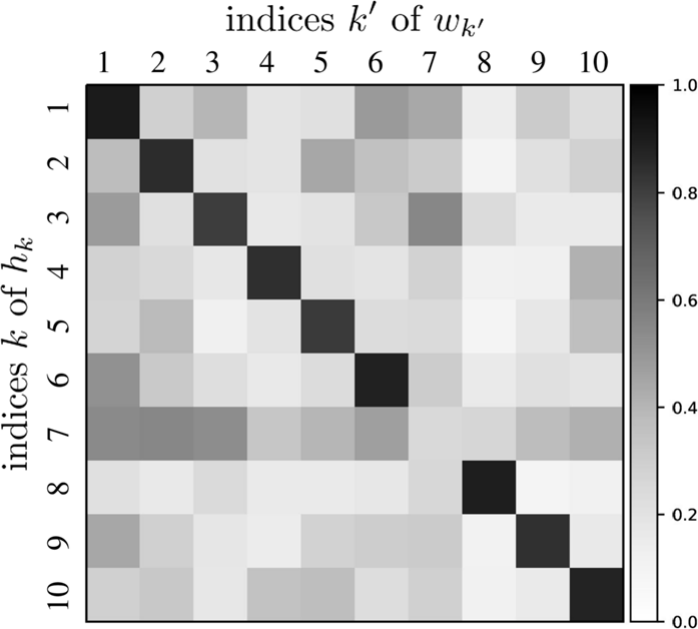
Cosine similarities between basis vectors. The vertical axis represents the indices of $h_{k}$, i.e., the *k*th row vector of *H*, and the horizontal axis represents the indices of $w_{k'}$, i.e., the $k'$th column vector of *W*. From top to bottom: $k=1,\ldots,K=10$; from left to right $k'=1,\ldots,K=10$. Scale bar displays the magnitude of cosine similarities between $h_{k}$ and $w_{k'}$

In all the other cases, we can observe that the pair of source and destination is located in mostly similar regions. To clarify this, Fig. 15 shows a matrix of cosine similarities between a basis vector of the source and a basis vector of the destination, where the cosine similarity of $h_{k}$ and $w_{k'}$ is calculated by

19 $$\begin{aligned} \frac{h_{k}\cdot w_{k'}}{ \Vert h_{k} \Vert \Vert w_{k'} \Vert }, \end{aligned}$$

where $h_{k}\cdot w_{k'}$ is the inner product of $h_{k}$ and $w_{k'}$, and $\|\cdot \|$ is the Euclidean norm of a vector. All the diagonal entries except for one are almost 1’s; that is, the *k*th basis vector $h_{k}$ is similar to the *k*th basis vector $w_{k}$ except for $k = 7$. These basis vectors correspond to basis vectors that have the previously mentioned geographically localized properties, and the similarities of pairs of basis vectors imply that both incoming and outgoing bank transfers for a local area have similar patterns.

Finally, we present the results of NMF with different values of *K*. To investigate the changes in the basis vectors which may occur according to *K*, we applied NMF to *V* with $K = 5, \dots , 15$. In all the cases, most of the basis vectors are geographically localized and form source and destination pairs that are similar to each other and correspond to bank transfers in local areas. All the basis vectors are localized for *K* less than 7, and a pair of basis vectors exists that corresponds to bank transfers between the largest city and local areas for *d* greater than or equal to 7. For all the examined values of *K*, the basis vectors correspond to either bank transfers in local areas or bank transfers between the largest city and other local areas.

## Conclusion

We studied an exhaustive list of bank accounts of firms and remittances from source to destination within a regional bank with a high market share of loans and deposits in a prefecture of Japan. By studying such a network of money flow, we could uncover how firms conduct the underlying economic activities as suppliers and customers from the upstream side to the downstream side of the money flow. We aggregated the remittances that occurred for each pair of accounts as a link during the period from March 2017 to July 2019 (i.e., approximately two and a half years), which comprises 30K nodes and 0.28M links. We found that the statistical features of the network are actually similar to those of a production network on a nationwide scale in Japan [3], but with greater emphasis on the regional aspects.

The bowtie analysis revealed what we refer to as a “walnut” structure in which the core and upstream/downstream components are tightly connected within the shortest distances, typically at a few steps. By quantifying the location of the individual account of a firm using the method of Hodge decomposition, we found that the Hodge potential of each node can describe the location in the entire flow of money from the upstream side to the downstream side, well characterized by the values of the potential. In particular, there is a significant correlation between the Hodge potentials and the net flows of incoming and outgoing money and links as well as the potentials and the walnut structure. This implies that we can characterize the net demand/supply of each node and decompose the flows into those due to the difference in potentials as well as divergence-free flows.

In addition, the network structure uncovered in this study can be used in predicting the default of firms. Particularly, because the financial information of small and medium-sized enterprises is often difficult to access, the credit risk management of banks will be improved by utilizing the information obtained from the network. Information on the network structure will be also useful in promoting the regional economy because the hubs of the GSCC can be firms playing a key role in the region. Studying the network of money flow can enable the prediction of what arises following an economic shock, which is essential in economic policymaking.

Furthermore, by using non-negative matrix factorization, we uncovered the fact that the entire flow can be considered as a combination of several significant factors. One factor has a feature whereby the remittance source is localized to the largest city in the region, while the destination is scattered. The other factors correspond to the economic activities specific to different local places, which can be interpreted as local activities of the economy.

We can consider several points that remain to be studied separately from the present work. While we aggregated the entire period in this paper, it would be interesting to determine how the network changes with time by examining the time-stamps recorded in every remittance. At time scales of days, weeks, and months, it is quite likely that there are intra-day, weekly, and seasonal patterns of activities. More interestingly, under mild changes in the booms and busts of the regional economy on a relatively long time scale, the economic agents might change their behaviors possibly by changing peers in the transactions. Alternatively, under sudden changes due to natural disasters or pandemics, the agents can change their usual patterns abruptly. In other words, these are important aspects of a temporally changing network. Capturing such dynamic patterns of remittances allows banks to forecast the timing of transactions and manage their liquidity more effectively.

In addition, further investigation of the aspect of money flow amounts is warranted in the sense that the dominant driving force likely comes from “giant players” who demand or supply a large amount of money. Moreover, it would be interesting to select them in a subgraph by choosing only links with flow amounts that are larger than a certain threshold. These topics will be studied in our future work.

## Figures and Tables

**Figure B.1:**
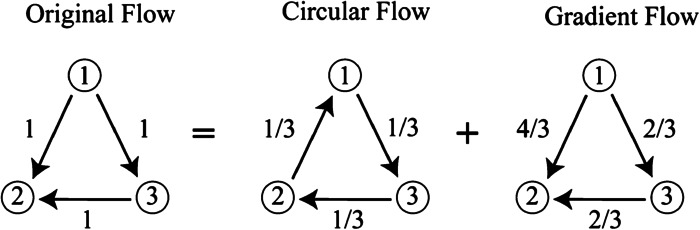
A simple example

**Figure B.2:**
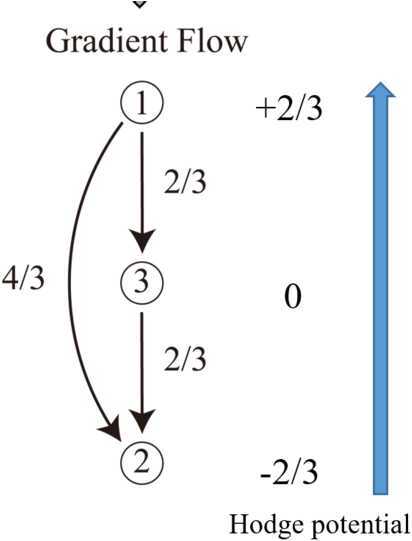
The illustration of the gradient flow network, given on the most right-hand side of Fig. B.1

**Figure B.3:**
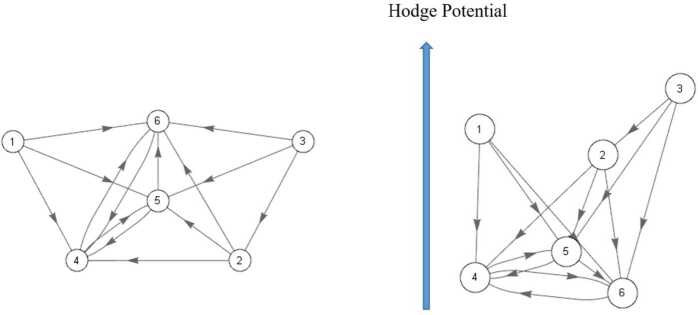
A sample network. On the left-hand side is visualization by the charge-spring visualization and on the right-hand-side is the visualization of the same network with the horizontal coordinates determined by the Hodge potential and the horizontal coordinate determined by the spring-charge method

**Figure B.4:**
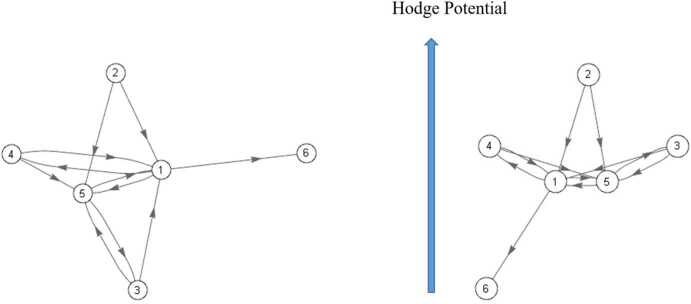
Another sample network, visualized in the same manner as in Fig. B.3

**Figure C.1:**
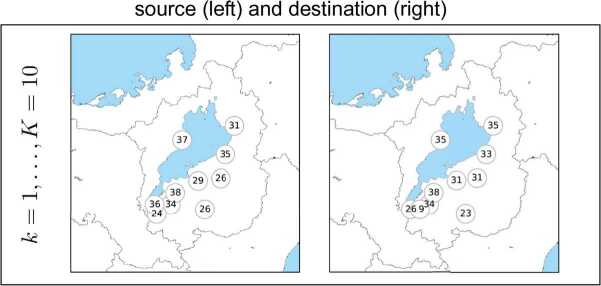
Circular areas corresponding to the basis vectors and proportions of the vector entries included in the circular areas. The left figure is for $w_{k}$, i.e., the basis vectors for sources, and the numbers are the levels of concentration, $\beta_{\max }(w_{k})$, while the right figure is for $h_{k}$, i.e., the basis vectors for destinations, and the numbers are $\beta_{\max }(h_{k})$, for $k=1,2,\ldots,K=10$

## Data Availability

The dataset is available in a collaborative scheme upon request to TT and YF at Shiga University.

## Appendix A: Network analysis

It would be beneficial to provide a set of stylized facts on the flow of money in our dataset of the bank accounts. In this appendix, we summarize the basic properties and statistics of the network.

A summary of network properties is given in Table A.1.

**Table A.1:** Summary of network properties

Property	Value	Note
Number of nodes	30,613	notation: *N*
Number of edges	280,864	notation: *M*
Density	0.0003	definition: *M*/*N*(*N* − 1)
Diameter	14	maximum of all shortest paths
Characteristic path length	4.2	average of all shortest paths
Number of multiple edges	0	by construction
Number of self-loops	0	by construction
Number of pairs of mutual edges	12,258	see (a)
Number of connected components	145	see (a)
Number of triangles	422,911	see (b)
Assortativity	−0.080	degree correlation; see (b)
Global clustering coefficient	0.035	see (b)

(a) A pair of mutual edges is such directed edges as *i*→*j* and *j*→*i* for a pair of nodes (*i*,*j*). (b) For the undirected version of the network, in which a pair of mutual edges, if any, is regarded as a single undirected edge.

Various centralities and properties for nodes are summarized in Table A.2. Definitions of these centralities are standard and some of them are recapitulated for convenience.

- Clustering coefficient of a node *i* is defined as $e_{i}/(k_{i}(k_{i}-1))$ where $k_{i}$ is the number of neighbors of *i*, and $e_{i}$ is the number of actually connected pairts between all neighbors of *i*.
- Connectivity of a node *i* is the number of its neighbors (i.e., $k_{i}$). Neighborhood connectivity of a node *i* is defined as the average connectivity of all neighbors of *i*.
- Average shortest path of a node *i* is the average length of a shortest path from *i* and any other node reachable from *i*. Let us denote it as $L_{\text{avg}}(i)$.
- Closeness of a node *i* is defined as $1/L_{\text{avg}}(i)$ (i.e., the reciprocal of the average shortest path of *i*).
- Eccentricity of a node *i* is the maximum non-infinite length of the shortest path between *i* and another node reachable from *i* in the network.
- Betweenness of a node *i*, $C_{\text{b}}(i)$, is defined by $C_{\text{b}}(i)=\sum_{s,t}{\sigma_{s,t}(i)}/{\sigma_{s,t}}$, where *s* and *t* are the starting and terminating nodes different from *i*, $\sigma_{s,t}$ is the number of shortest paths from *s* to *t*, and $\sigma_{s,t}(i)$ is the number of shortest paths from *s* to *t* such that *i* is on the path.

**Table A.2:** Centralities and properties of nodes

Property	Min.	25%Q	Median	75%Q	Max.	Avg.	Note
Degree	1	1	4	17	2245	18.3	total
In-degree	0	1	2	6	1464	9.17	
Out-degree	0	0	1	8	2245	9.17	
Clustering coeff.	0.0	0.0	0.015	0.056	1.0	0.061	see def.
Neighborhood Connectivity	1.0	53.0	112.6	194.0	2245.0	182.8	see def.
Avg. shortest path length	0.0	0.0	3.50	4.17	8.80	2.28	see def.
Closeness	0.0	0.0	0.21	0.25	1.0	0.18	see def.
Eccentricity	0	0	9	10	14	5.40	see def.
Betweenness	0.0	0.0	0.0	4.71 × 10^3^	2.24 × 10^7^	3.88 × 10^4^	see def.

25%Q and 75%Q are respectively 1st and 3rd quantiles.

## Appendix B: Hodge decomposition

As explained in the main text, Hodge decomposition plays an essential role in studying the network structure, by allowing the researchers to quantitatively order the nodes according to their connectivity to other nodes.

One way to understand it to study some simple examples. One of the most simple but nontrivial one is illustrated in Fig. B.1. The network illustrated on the most left-hand-side (“Original Flow”) is made of the three nodes with the given flow. The flows are decomposed to “Circular flow” and “Gradient Flow” as are illustrated. Sum of the two flows are equal to the original flow: For example, from the node no.1 to the node no.2, circular flow is $-1/3$ (as it is $+1/3$ in the other direction) and the gradient flow is $+4/3$, which adds up to 1, the value of the original flow. Also, the gradient flow satisfies the property (7). Furthermore, the gradient flow satisfies Eq. (8) with all the weights equal to one ($w_{ij}=1$) and the Hodge potential $(\phi_{i})=(+2/3, -2/3, 0)$. Figure B.2 shows the visualization of this network with the use of the Hodge potential $(\phi_{i})$ as vertical coordinate. In this illustration it is straightforward to see that gradient flows are equal to the difference of the Hodge potentials of the relevant nodes.

Figure B.3 and Fig. B.4 are simple and more illustrative examples, where all flows are of strength 1 as in the first example. In both Figures, on the left panel is the visualization of the whole network by using the spring-charge method, and on the right panel is the visualization of the same network with the vertical coordinate determined by the Hodge potential and the horizontal coordinate determined by the spring-charge method.

In Fig. B.3, the nodes are placed in a left-right symmetric manner on the left panel, although the links do not have the same symmetry. The nodes no.1 and no.3 are placed in same vertical position. If one used the total out-flow as a measure of the rank, they would be placed just like this, as both of them have the total out-flow equal to three. The right panel, however, shows a different picture: Nodes no.1 and no.3 are placed at different heights, due to the difference in their Hodge potential, which again is due to the difference in the way they are connected to other nodes.

The example in Fig. B.3 shows the power of the Hodge decomposition in a different manner: On the left-panel, we do not see any symmetry and the roles of the nodes are not apparent. On the contrary, the right panels shows the left-right symmetry except for the node no.6. Nodes no.1 and no.5 plays very similar role in this network, the only difference being that no.1 is connected to no.6. Same is true for the nodes no.4 and no.3. Without the use of the Hodge decomposition this fact is rather difficult to see.

As seen in these examples, the Hodge potential plays an important role in clarifying the whole structure of the network.

## Appendix C: NMF basis vectors: spatial concentration

As we showed in the main text, the NMF basis vector’s components are concentrated in small geographical regions, because of the fact that the vector components have peaks at specific locations in most cases. In this appendix, we shall quantify the concentration and the peaks, and show results.

Recall that the entire region was divided into *L* by *L* small squares $R_{\ell}$ ($\ell=1,2,\ldots,L^{2}$) in a lattice grid, where we set $L=100$. A basis vector *v*, which is either a column vector $w_{k}$ of *W* or a row vector $h_{k}$ of *H*, has its components at the indices *ℓ*, each of which corresponds to a different location $R_{\ell}$. Because all components of the basis vector *v* are non-negative by construction of the NMF, the *v*’s vector components can be represented as a heatmap in the geographical region. This representation was actually used in Fig. 11, Fig. 12, Fig. 13, and Fig. 14.

Let $r_{\ell}$ be the center of the square $R_{\ell}$, and $C_{\ell }$ be a circle centered at $r_{\ell}$ with a certain radius. We choose the radius as 5 km in order to avoid overlapping of circles. The choice of the radius is not essential because the circle is not related to the NMF and is used only for quantification of geographically localized structure. For a vector *v* and a circle $C_{\ell}$, let us define

C.1 $$ \beta(C_{\ell},v)= \frac{\sum_{\{\ell' \mid r_{\ell'}\in C_{\ell}\}} v_{\ell'}}{\sum_{\ell''=1}^{L^{2}} v_{\ell''}} . $$

This quantity represents how the components of *v* are concentrated in the circle $C_{\ell}$. Then by finding and calculating

C.2 $$\begin{aligned} &C_{\max }(v)= \mathop{\operatorname{arg\,max}} _{\{C_{\ell }\mid\ell=1, \ldots,L^{2}\}} \beta(C_{\ell},v) , \end{aligned}$$

C.3 $$\begin{aligned} &\beta_{\max }(v)= \max_{\{C_{\ell }\mid \ell=1,\ldots,L^{2}\}} \beta(C_{\ell},v) , \end{aligned}$$

one can identify the circle $C_{\max }(v)$ that maximizes the concentration $\beta(C_{\ell},v)$, and can quantify the level of concentration by $\beta_{\max }(v)$.

In all the cases (except $h_{k}$ for $k=7$), the basis vectors $w_{k}$ and $h_{k}$ have such peaks meaning that the corresponding sources and destinations are well localized in the geographical region. The circles shown in the figures of Fig. 11, Fig. 12, Fig. 13, and Fig. 14 are given by this procedure.

Figure C.1 shows the levels of concentration $\beta_{\max }(v)$ for all the pair of $w_{k}$ and $h_{k}$ depicted together as 10 circles corresponding to $k=1,2,\ldots,K=10$ different NMF components. The numbers in the circles are the levels of concentration. The levels of concentration are more than 23% except for one basis vector in both figures of the source and destination; therefore, most basis vectors of bank transfers are localized geographically. Since the positions of the circular areas are around the centers of cities, geographically localized properties are thought to reflect the economic activity in those local areas. This is how we identified city names in each boxes of Fig. 11, Fig. 12, Fig. 13, and Fig. 14. The single exception is the basis vector $h_{k}$ for $k=7$, for which the level is only 9%. This means that the destinations are spread over the prefecture of Shiga and also Kyoto.
